# Regional Variation in Human Exposure to Persistent Organic Pollutants in the United States, NHANES

**DOI:** 10.1155/2015/571839

**Published:** 2015-12-29

**Authors:** Wendy A. Wattigney, Elizabeth Irvin-Barnwell, Marian Pavuk, Angela Ragin-Wilson

**Affiliations:** Division of Toxicology and Human Health Science, Agency for Toxic Substances and Disease Registry, 4770 Buford Highway, Atlanta, GA 30341, USA

## Abstract

We examined serum levels of persistent organic pollutants (POPs) among geographical regions of the United States as defined by the US Census Bureau. National Health and Nutrition Examination Survey (NHANES) data for adults aged 20 years and older are presented for selected survey periods between 1999 and 2010. From NHANES 1999 through 2004, dichlorodiphenyldichloroethylene (DDE) concentration levels were consistently higher among people living in the West than in the Midwest, Northeast, or South. In 2003–2010, perfluorinated compound concentrations tended to be highest in the South. The sum of 35 polychlorinated biphenyls (PCBs) congeners was significantly higher in the Northeast [GM: 189; 95% CI: 173–204 ng/g lipid] than the remaining regions. The regional differences in higher body burdens of exposure to particular POPs could be attributed to a variety of activities, including region-specific patterns of land use and industrial and agricultural chemical applications, as well as different levels of regulatory activity.

## 1. Introduction

Biomonitoring to assess body burden levels of environmental toxicants in humans has become a cornerstone of environmental public health efforts [1]. Potentially hazardous chemicals are released into the environment from a wide range of sources including industrial discharges, agricultural run-off, leachate from landfills, human waste disposal, and even the use of household products. Persistent toxic chemicals do not easily degrade in the environment and can bioaccumulate in animals and people after repeated exposure to contaminated air, water, sediment, soil, and food. Many persistent, bioaccumulative, and toxic pollutants were banned and phased out of commerce decades ago, but because they are persistent they still pose a risk to people throughout the United States [2]. International negotiations under the United Nations Environmental Programme have led to the development of legally binding criteria to regulate the ongoing exposure to these “legacy” contaminants, particularly persistent organic pollutants (POPs) [3]. Numerous contaminated sites, ranging from chemical dumpsites to accidental industrial spills, have posed health risks in communities across the nation.

In the general population the main source of exposure to “legacy” POPs, such as dioxins, polychlorinated biphenyls (PCBs), and persistent pesticides, is food made from plants and animals in which these substances have accumulated [2, 3]. These substances can migrate into the soil and sediment that support nutritional resources for wild or farmed animals, including resources used to manufacture animal feeds. Exposure to more recent POPs, such as polybrominated diphenyl ethers (PBDEs) and perfluoroalkyls (PFCs), is primarily due to their use in consumer products such as flame retardants and surfactants, respectively. Consuming fish and wildlife caught from locally contaminated ecosystems increases exposure risk in certain subpopulations. Breathing contaminated air and contact with contaminated dust, sediment, soil, or water are also pathways of exposure.

Human biomonitoring studies have shown that essentially all people have low levels of multiple environmental chemicals in their bodies [1]. The presence of an environmental chemical in a person's body does not necessarily indicate that the chemical has caused or will cause a disease. Epidemiologic investigations help determine the human health impact of environmental chemicals at varying exposure levels. Studies indicate that elevated levels of some persistent toxic chemicals are associated with adverse reproductive outcomes, developmental disabilities, endocrine disorders, and neurological effects [4–12]. Other cross-sectional studies have found that elevated body burdens of PCBs, lead, and mercury are associated with unexplained liver disease [13, 14]. Additionally, recent investigations found an association between PFCs and thyroid function in US adults [15, 16].

Large-scale biomonitoring programs monitor the prevalence of exposure to environmental toxins and help inform environmental policy and practice. The National Health and Nutrition Examination Survey (NHANES), conducted by the National Center for Health Statistics, provides data to assess the health and nutrition status of the US population [17, 18]. Laboratory analysis of biological samples from NHANES participants, conducted by the National Center for Environmental Health (NCEH), provides an ongoing assessment and overview of the exposure of the US population to environmental chemicals. NCEH's* Fourth National Report on Human Exposure to Environmental Chemicals (Fourth Report) *provides geometric means and percentiles of environmental chemicals in people by 2-year NHANES data cycles and descriptive statistics by age group, gender, and race/ethnicity [19, 20]. The* Fourth Report* also provides summary information about each chemical, including uses, sources of exposure, effects in animals or humans, and comparative levels from other studies.

**Figure 1:**
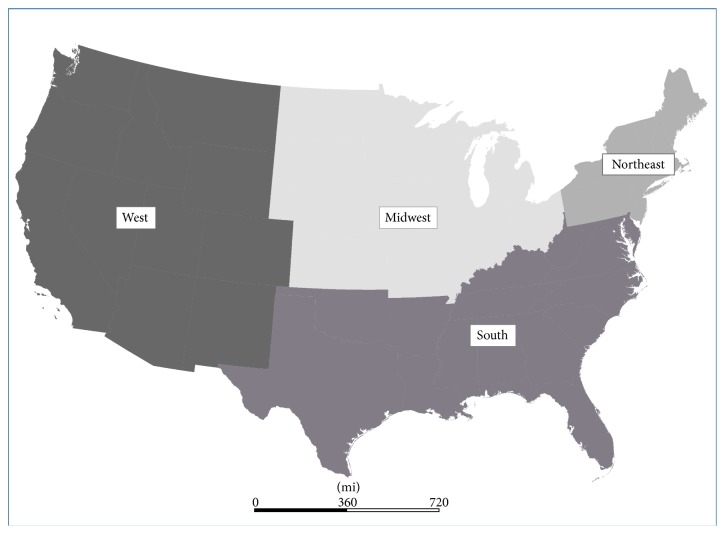
Census regions of the United States.

The primary purpose of the current report is to estimate population body burden levels of selected POPs within US Census regions (Figure 1: Northeast, Midwest, South, and West) [21]. NHANES data requires weighted analysis based on a complex, stratified sampling strategy which allows populations estimates within these broad US geographic subdivisions. As such, this division of states has been used to classify NHANES environmental exposure data regionally, for example, blood mercury concentrations [22]. NHANES data does not support unbiased population estimates for other US geographic subdivisions. Contrasting the results obtained from the NHANES data by US region might reveal patterns of exposure for legacy and emerging toxic chemicals by geographical region. We speculate that access to education, health care, employment, and other lifestyle factors vary substantially by region, as does the historical level of production, use, and disposal of many chemicals. For site-specific exposure assessments, regional estimates of environmental toxins provide reference values more localized than national NHANES data. Regional differences in POPs, or lack thereof, also can inform physiologically based pharmacokinetic modeling used in health risk assessments [23]. A lack of substantial regional variation supports the use of NHANES national estimates as referent values. Identifying regional patterns in exposure to POPs can be preliminary to focusing searches on differences in health effects. 

## 2. Material and Methods

### 2.1. Data Source

We used NHANES data for selected survey periods between 1999 and 2010. The survey incorporates a complex, stratified, multistage, probability-cluster design to examine a nationally representative sample of about 5,000 persons annually and collects biological specimens for environmental chemical analysis. The primary sampling unit is the selection of counties from a sampling frame that includes about 3,000 counties in the contiguous United States. The NHANES national sample included 12 counties in 1999 and 15 counties in each year from 2000 through 2010. NHANES releases data in two-year cycles in order to provide sufficient sample sizes to obtain stable national estimates and to focus on health and nutrition measurements to capture emerging needs. In each cycle, selected environmental chemicals are measured in blood, serum, and urine from a random subsample (i.e., 1/2 sample or more commonly 1/3 sample) of participants within specific age groups [24]. The* Fourth Report *provides detailed information on the selection of subsamples for chemicals or chemical groups [19]. NCHS's Ethics Review Board approved NHANES protocols and all participants aged 18 years and older provided written informed consent.

To protect the confidentiality of NHANES participants, public use data do not include location parameters. However, the NCHS developed the Research Data Center (RDC) to provide a mechanism that permits researchers access to restricted data. We worked with the RDC to create a dataset that includes an indicator variable for US Census region for selected chemicals.

### 2.2. Statistical Methods

The NHANES sample design is based on a complex multistage strategy. The NCHS provides weights for the analysis of NHANES data to account for oversampling, possible nonresponse bias, and poststratification to US Census Bureau estimates of the US population [25]. Because our estimates are based on US Census regions, we used unmasked stratum and primary sampling units provided by the NCHS RDC to designate the complex sample design in our analyses.

Geometric means (GM), percentiles, and corresponding 95% confidence intervals (CIs) were calculated following NCHS recommended methods which are provided in NHANES data analysis tutorials and the* Fourth Report *[19, 24–26]. Ninety-five percent CIs around the GM were calculated by adding and subtracting the following value: the product of a Student *t*-statistic (with degrees of freedom equal to the number of primary sampling units minus the number of strata) and the standard error of the weighted GM estimate. SUDAAN was used with the sample weights to calculate variance estimates via the Taylor series linearization method. We estimated the 50th and 90th percentiles and corresponding 95% CIs using methods which are described in the* Fourth Report (Appendix A) *[19]. We used the statistical software packages SAS version 9.3 (SAS Institute Inc., 2002–2011) and SUDAAN (SUDAAN Release 11.0, 2012) [27].

The NHANES region-specific analysis is likely affected by small numbers of degrees of freedom, particularly for a single 2-year cycle. Subdomains within the national sample will likely have higher variances of the estimated standard errors, affecting inferences. When possible, we combined NHANES 2-year cycle data to obtain more precise standard error estimates and CIs. Supplemental Table 1 (in Supplementary Material available online at http://dx.doi.org/10.1155/2015/571839) provides a summary of the available NHANES cycles for the chemical data included in this report. We followed NCHS guidelines for the derivation of sample weights for combined NHANES survey cycles. The RDC suppresses information on the number of primary sampling units, degrees of freedom included in our analyses. We do know that the region-specific degrees of freedom for even combined cycles tended to be slightly less than 12. The NHANES guidelines recommend at least 12 degrees of freedom when calculating estimates for subgroups of interest within the total NHANES population [25].

The chemicals included in the report were all measured in serum. For PCBs, dioxins, and organochlorine pesticides, lipid-adjusted concentrations are used in the data analyses. These compounds are lipophilic and the lipid-adjusted concentration reflects the amount stored in body fat. We analyzed data by the four US Census regions for adults aged 20 years and older. For chemical selection, we included chemicals that had a detection frequency of at least 60%. Specifically, we examined selected non-dioxin-like PCBs, selected PBDEs, 1,2,3,6,7,8-hexachlorodibenzo-p-dioxin (HxCDD), 1,2,3,4,6,7,8-heptachlorodibenzo-p-dioxin (HpCDD), and DDE and four PFCs (PFOA, PFOS, PFNA, and PFHxS). In addition, we calculated total PCB concentration (sum of 35 PCB congeners on a lipid-adjusted basis) using methods described by Patterson Jr. et al. [3]. Dioxin-like total toxic equivalency (TEQ) was calculated based on WHO 2005 toxic equivalency factors for polychlorinated dibenzo-p-dioxins, dibenzofurans, coplanar biphenyls, and mono-ortho-substituted biphenyls as described by Patterson Jr. et al. [28]. For TEQ estimates, we present only the 90th percentile because the detection frequency was less than 40% for some congeners included in the calculation. The 50th percentile is likely to be biased when the percentage of results below the detection limit is near or above 50% [29].

We examined regional differences in levels of POPs using analysis of covariance adjusting for age (years), smoking status (current smoker, former smoker/never smoked), gender (male, female), and race/ethnicity (Mexican American, non-Hispanic White, non-Hispanic Black, and other). For multiple regression models, we calculated least-square means based on log_10_-transformed analyte values using the REGRESS procedure in SUDAAN. We treated age as a continuous variable and smoking status, gender, and race/ethnicity as categorical variables. For each analyte, we ran the multiple regression model with alternate regions defined as the referent to examine all pairwise regional comparisons of least-squared geometric means with a *p* < 0.01 level of statistical significance. We used an alpha level of 0.01 rather than conventional 0.05 to account for multiple comparisons. Specifically, 0.01 corresponds roughly to 0.05 divided by 6, where 6 is the total number of pairwise comparisons for 4 regions.

## 3. Results

To protect the privacy of individuals, the RDC cannot release information regarding the number of counties or individuals included in each region. As such, sample sizes are not reported. To minimize repeated use of words, we refer to concentration levels among people living in a region as “in the region.” For example, “in the West” refers to concentration levels among people living in the West.  Table 1 contains descriptive analysis of NHANES 2003-2004 lipid-adjusted levels of selected non-dioxin-like PCBs among people living in the four Census regions. The geometric mean serum concentration of PCB 28 was higher in the West (GM: 5.4, 95% CI: 4.5–6.4 ng/g lipid) than in the Northeast (GM: 4.1, 95% CI: 3.4–4.9 ng/g lipid) but not significantly different from the South or Midwest; PCB 52 and PCB 101 serum concentrations were higher in the West (PCB 52 GM: 3.5, 95% CI: 3.1–3.9 ng/g lipid; PCB 101 GM: 2.1, 95% CI: 1.8–2.4 ng/g lipid) than all other regions; PCB 74, PCB 99, and PCB 206 serum concentrations were higher in the Northeast (PCB 74 GM: 6.6, 95% CI: 4.9–8.3 ng/g lipid; PCB 99 GM: 6.3, 95% CI: 4.4–8.3 ng/g lipid; and PCB 206 GM: 3.5, 95% CI: 2.4–4.5 ng/g lipid) than all other regions; and, PCBs 196/203 levels in the Northeast (GM: 4.3, 95% CI: 2.6–6.0 ng/g lipid) and West (GM: 3.4, 95% CI: 1.8–5.0 ng/g lipid) were higher than levels in the Midwest (GM: 3.2, 95% CI: 2.0–4.3 ng/g lipid) and South (GM: 3.1, 95% CI: 2.4–3.8 ng/g lipid). PCBs 138 and 158 and PCB 153 were examined over the available 2001–2004 cycles and were significantly higher in the Northeast than the other regions. The GM of PCB 138/158, NHANES 2001–2004, in the Northeast was 31–59% higher than the GM from the remaining three regions (Table 2). In 2001–2004, regional differences were not indicated for serum concentrations of the dioxin HxCDD; and HpCDD was lower in the West (HpCDD GM: 27.1, 95% CI: 24.4–29.7 pg/g of lipid) than in the Midwest (HpCDD GM: 35.9, 95% CI: 27.7–44.1 pg/g of lipid) and South (HpCDD GM: 34.1, 95% CI: 29.6–38.7 pg/g of lipid) (Table 2). Regional differences were determined after adjusting for smoking status, gender, race/ethnicity, and age.

As shown in Table 3, NHANES 1999–2004 survey data indicate that the pesticide metabolite DDE measured in serum was significantly highest in the West (GM: 476, 95% CI: 390–563 ng/g lipid) compared with all other regions. In 1999–2004, serum concentrations of DDE were also significantly higher in the South (GM: 311, 95% CI: 262–360 ng/g lipid) than in the Northeast (GM: 247, 95% CI: 211–284 ng/g lipid) and Midwest (GM: 232, 95% CI: 201–263 ng/g lipid).

Table 4 presents NHANES 2003-2004 levels of selected PBDEs in serum by region. The PBDE congeners analyzed include BDE 28, BDE 47, BDE 100, and BDE 153. The South and West had significantly higher concentrations of these PBDE congeners compared with the Northeast. PBDE serum concentration levels in the West were consistently the highest, particularly at the 90th percentile although the exact 95% CIs were notably wide for BDE 47 and BDE 100 levels (BDE 47 90th percentile: 171 ng/g of lipid, 95% CI: 70.0–589 ng/g of lipid; BDE 100 90th percentile: 34.5 ng/g of lipid, 95% CI: 8.4–158 ng/g of lipid).

**Figure 2:**
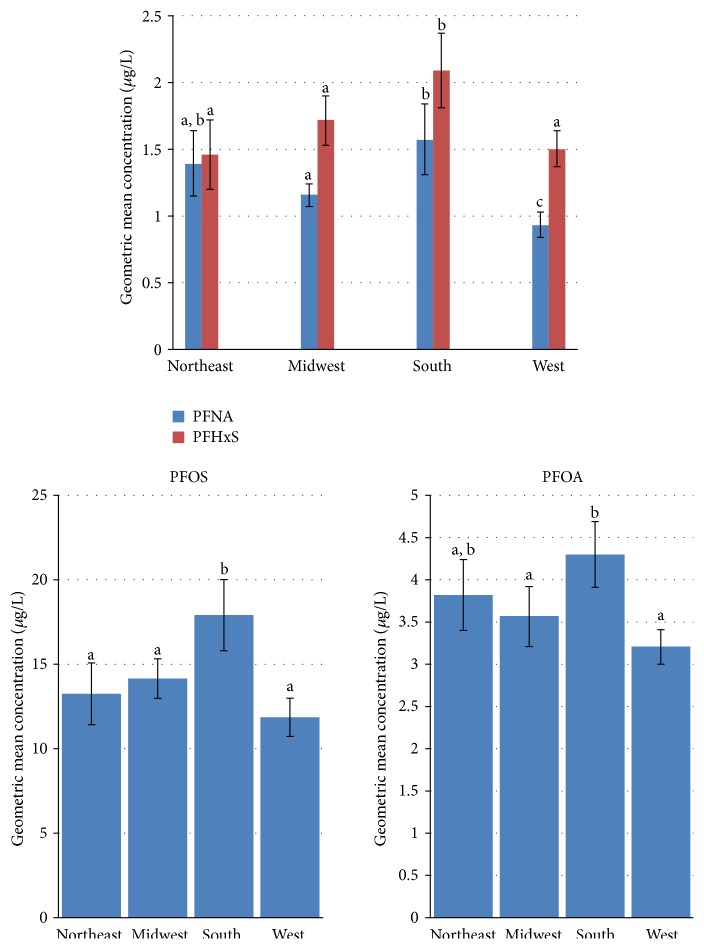
Geometric mean serum concentrations (in *µ*g/L) of PFCs by US Census region, NHANES 2003–2010. The error bars represent the 95% confidence interval. Within survey periods, regional means with the same letter superscript are not significantly different from each other based on analysis of covariance adjusting for age, smoking status, gender, and race/ethnicity.

We examined regional PFC levels in NHANES data for 2003–2010 (Figure 2). PFNA concentrations were significantly lower in the West (GM: 0.9 *μ*g/L, 95% CI: 0.8–1.0 *μ*g/L) than in the South (GM: 1.6 *μ*g/L, 95% CI: 1.3–1.8 *μ*g/L), Midwest (GM: 1.2 *μ*g/L, 95% CI: 1.1–1.2 *μ*g/L), or Northeast (GM: 1.4 *μ*g/L, 95% CI: 1.2–1.6 *μ*g/L). Regional comparisons in PFHxS and PFOS serum concentrations showed significantly higher levels in the South compared with all other regions. Perfluorooctanoic acid (PFOA) was significantly higher in the South (GM: 4.3 *μ*g/L, 95% CI: 3.9–4.7 *μ*g/L) only in comparison to the West (3.2 *μ*g/L, 95% CI: 3.0–3.4 *μ*g/L). Supplement Table 2 presents the geometric means and 50th and 90th percentiles of serum PFC concentrations for the US population ages of 20 years and older by geographic area.

In 2003-2004, total PCB concentration was significantly higher in the Northeast (GM: 189 pg/g of lipid, 95% CI: 173–204 pg/g of lipid) than all other regions. Geometric mean total TEQ levels measured in 2003-2004 were lower in the West compared to the Northeast, Midwest, and South. The 90th percentile TEQ levels in the Northeast (35.5 ng/g of lipid) and Midwest (35.3 ng/g of lipid) were notably higher than levels in the South (29.7 ng/g of lipid) and West (28.2 ng/g of lipid) (Table 5).

## 4. Discussion

A number of factors can influence levels of exposure to persistent toxic chemicals and subsequent body burden levels, including occupation, nutrition, age, time of exposure along with residence time in the human body, exposure concentration and duration of exposure, smoking status, race/ethnicity, and gender [3, 30–32]. NHANES environmental chemical data provide descriptive statistics for the total US population and by age group, gender, and race/ethnicity. The NHANES data also provide useful information such as the 95th percentile which can serve as a reference value for determining unusually high levels in separate public health investigations [17, 20, 33, 34]. This study examines levels for environmental chemicals by US Census region based on weighted analysis so that the descriptive statistics are representative of each region. Chemical and toxicological information about the analytes included in the current report can be found in the Agency for Toxic Substances and Disease Registry (ATSDR) Toxicological Profiles [35] and the* Fourth Report on Human Exposure to Environmental Chemicals *[19, 20]. These resources provide descriptive information about each chemical or chemical group including uses and sources of exposure.

In the United States, commercial production of PCBs began in the late 1920s and ceased in the late 1970s. Variation in PCB usage and manufacturing led to variation in human exposure over time and in different geographic locations [36]. Because of low biological degradability, PCBs persist in contaminated sediment and continue to cause concern for human health. For example, PCBs were manufactured in Anniston, Alabama between 1929 and 1971, and PCB concentrations measured in residents three decades later are two to three times higher than NHANES data for comparable age and race groups [37]. In our report, analysis showed that several non-dioxin-like PCBs concentrations tended to be higher in the Northeast (PCBs 74, 99, 196/203, 206, 138/158, 153, and 196/203) than in the Midwest or South. Some occupationally related PCB congeners, for example, PCB 74, were still elevated in former capacitor manufacturing workers after almost three decades [38]. The sum of 35 PCB congeners was significantly higher in the Northeast than all other regions.

Dichlorodiphenyltrichloroethane (DDT) was a widely used broad-spectrum insecticide that the United States banned in 1972. Some countries still use DDT primarily for malaria control. In the environment and in the body, DDT converts to DDE and other metabolites or chemicals. Mean DDE serum levels in NHANES II, 1976–1980, participants were on average five times higher than levels presented in our report using NHANES 1999–2004 data [39]. Persons living in the South or West had significantly higher geometric mean DDE serum levels in the 1976–1980 survey, similar to the regional variation seen in our analysis of 1999–2004 NHANES data. As noted in Stehr-Green's report [39], persons living in the South and West appear to be at greater risk of DDE exposure perhaps due to regional patterns in land use for agriculture and associated increased use of pesticides. Although serum concentrations of DDE in the US population have declined since the late 1970s, people in the US continue to be exposed to DDT/DDE primarily by eating meat, fish, and dairy products.

An assessment of background exposures of the general US population to dibenzo-p-dioxins (PCDDs) noted a decline in TEQ values from the 1990s to the 2000s which is likely attributed to reductions in TEQ concentrations in pork, poultry, and milk [40]. In our report, people living in the Midwest and South during the NHANES 2001–2004 survey period had higher concentrations of the chemical HpCDD compared to the West. The University of Michigan Dioxin Exposure Study (UMDES) found mainly background levels of PCDDs and polychlorinated dibenzofurans (PCDFs) in one of the largest population-based human exposure studies to these substances [41]. Residents living closer to a point-source production facility in Midland, MI, and people living on properties used for animal and crop productions in the 1940s through 1960s had elevated concentrations of PCDDs and PCDFs in the UMDES study [41]. The regional distributions of NHANES 2003-2004 total TEQ concentrations showed considerable overlap; however, levels at the 90th percentile tended to be higher in the Northeast and Midwest than in the South or West.

PBDEs are common synthetic flame retardant chemicals produced since the 1970s. The concentrations of PBDE congeners in mothers' milk were found to be at least 10 times greater in the US than in Sweden in the early 2000s [42, 43]. PBDEs were first analyzed in NHANES samples in the 2003-2004 survey. BDE 47 was detected in almost all participants and BDE 28, BDE 100, and BDE 153 were detected in at least 60% of participants. Among these PBDE congeners, BDE 47 had the highest serum concentrations with higher levels in children aged 12–19 years, Mexican Americans, and males [44]. Longitudinal analyses of NHANES 2003–2008 data show that PBDE serum concentrations did not decrease during this time period [45]. Commercial PentaBDEs were discontinued in 2004; and commercial DecaBDEs, the most widely used PBDE globally, were scheduled to be phased out of production in 2013 [45]. In our report, BDE 28, BDE 47, BDE 100, and BDE 153 levels were highest in the West and lowest in the Northeast. Elevated PBDE serum levels in the western part of the nation compared to the Northeast have also been reported in other studies. Horton et al. [46] found lower levels of PBDEs in a 2009-2010 New York City cohort than levels in an analogous cohort living in California. Zota et al. [47] examined the 2003-2004 NHANES data and found that California residence had serum ∑PBDE nearly 2-fold higher than other North American regions. These findings may be explained by California enacting stringent flammability standards for furniture manufacturing in the 1970s. Until the Department of Consumer Affair's Technical Bulletin 117-2013 on requirement for testing materials in upholstered furniture was introduced in 2013, the standards in California for flammability were the highest in the US [48].

PFCs are used to make heat resistant coatings for a wide variety of consumer products such as cookware, clothing, furniture, packaging, and electrical insulation. The manufacturing of PFOS, an earlier PFC, came under scrutiny in the late 1990s after toxicity data indicated health risks. The United States phased out PFOS production in 2000 [49]. The 3M facility in Minnesota was the primary producer of PFOS, raising concern about human exposure from contamination of the Mississippi River and surrounding areas [50]. The Ohio River Valley community study provides evidence of a large population exposure to elevated levels of PFOA mainly in drinking water [50]. Generally, global production of PFCs continues, such as PFOA, although with increased efforts to limit emissions. PFCs do not easily break down or degrade in the environment; they have been detected in coastal and ocean waters, marine and land animals, and humans [19]. PFCs are among the new chemicals added for the NHANES 2003-2004 survey period. The four PFCs included in our report (PFNA, PFHxS, PFOA, and PFNA) are detected in nearly all NHANES serum samples indicating that exposure to these chemicals was widespread. PFOS concentrations were higher in the South compared with the other three Census regions. An earlier report on trends in exposure to PFCs in the US population shows a sharp continuous decline in PFOS concentrations in NHANES 2-year survey periods from 1999 to 2008 [49]. The marked decrease in PFOS concentrations is consistent with the discontinued production in the US in 2002.

Legislative policies to ban bioaccumulative chemicals suspected to pose adverse health effects and restoration efforts to clean up contaminated sediments and soils in areas of concern are well-known strategies to reduce and eventually eliminate exposure to persistent toxic substances [51]. Programs that monitor aquatic species, food sources, and people provide evidence of progress towards remediating legacy contamination. Fish monitoring data show continued long-term annual declines (after 1990) for PCBs in fish caught in Great Lakes open waters [52, 53]. Researchers who conducted a 2000-2001 national survey on toxic pollutants in the US milk supply estimated that the average daily intake of pesticides, dioxins, and metals from total milk fat ingestion has decreased compared with intake of chemicals estimated in the 1996 survey [54]. Human biomonitoring data also show decreases over time in body burdens of legacy toxic pollutants, particularly in children and young adults [45, 49, 55]. Most levels of legacy contaminants increase with increasing age, indicating that levels are the result of cumulative past exposures [3, 19]. Beginning with sera collected in NHANES 2003-2004, advances in laboratory methods allowed the measurement of low levels of non-dioxin-like PCBs in the US population. NHANES 2003-2004 estimates of PCDD/F levels (dioxins and furans) are generally lower than levels documented in surveys of selected populations in previous decades [55]. PFOS concentrations in the US population decreased from NHANES survey period 1999-2000 to 2007-2008; however, concentrations of other detectable PFCs increased over this time period [49].

The apparent regional differences in higher body burdens of exposure to particular POPs could be attributed to a variety of exposure conditions, including region-specific patterns of land use and industrial and agricultural chemical applications, as well as different levels of regulatory activity. For instance, people living in the South and West in 1999–2002 appeared to be at greater risk of pesticide exposure which could be due to a higher concentration of agriculture and farms which require the use of pesticides. Additionally, the regional variations in phased-out organochlorines could be attributed to differences in consumption rates of food items with a higher content such as seafood and animal fat. Regional patterns of exposure to other chemicals may reflect concentration of related industrial activity within the area. Numerous industrial facilities (i.e., capacitor manufacturing plants, automakers' foundry plants) and waste sites that historically used and in many cases still contain PCBs were located in the Northeast United States and were studied extensively [38, 56–58]. It is possible, but not likely taking into account the sampling scheme of NHANES, that occupational exposures and residents living in the vicinity of those sites may have contributed to higher average PCB concentrations seen in this region.

Protecting human health from the adverse effects of legacy chemical contaminants and chemicals of emerging concern continues to be an important focus of current surveillance and monitoring programs. However, numerous natural and synthetic chemicals are widely used in industry and our daily lives. Monitoring ecosystems has identified chemicals of emerging concern, and ongoing advances in analytical chemistry allow detection of chemical compounds often present only at trace levels. Detection of newly identified chemicals in the environment requires risk assessment studies to understand the extent to which these chemicals pose a threat to the ecosystem and human health [59]. Contaminants of emerging interest include current use pesticides, pharmaceuticals, brominated flame retardants, bisphenol A, phthalates, perfluorinated surfactants, and synthetic musk. Monitoring the complex mixture of legacy contaminants and emerging chemicals in ecosystems and humans presents a public health challenge that requires collaboration among local, state, and federal agencies and researchers.

NHANES serves an essential role in establishing population-based reference ranges for environmental chemicals and in collecting information needed for epidemiological research. However, NHANES has limitations; the survey alone cannot inform on area-specific exposures that might lead to public health actions. State-based public health biomonitoring programs could better assess human exposure to environmental chemicals [60]. Biomonitoring data will allow state public health officials to reduce or eliminate exposure to certain environmental chemicals by helping to identify at risk subpopulations within their jurisdiction and assess the effectiveness of public health actions to reduce harmful exposures [60]. Biomonitoring findings can also be integrated into the development and implementation of chemical use policy.

The analysis presented in this report is based on sampling that uses a relatively small number of counties as the primary sampling unit within the broader US regions. As such, we do not know which areas of the US regions are represented. Additionally, NCHS suppresses information about which counties were selected, the number of participants within each region, and the number of primary sampling units (i.e., degrees of freedom) that are included in the analysis. The reliability of our region-specific NHANES estimates may be unstable due to the small number of primary sampling units. These sampling factors and sample size limitations may lead to bias in our regional comparisons.

## Supplementary Material

Supplemental Table 1 provides a summary of the NHANES survey years selected based primarily on availability for each environmental chemical in the current report. Supplemental Table 2 provides the geometric mean concentrations and selected percentiles (in micrograms per liter) of serum perfluorononanoic acid (PFNA), perfluorohexane sulfonic acid (PFHxS), perfluorooctanoic acid (PFOA), and perfluorooctane sulfonic acid (PFOS) for adults ages 20 years and older by U.S. census region for NHANES 2003-2010. These data supplement the regional comparisons based on geometric means that are presented in Figure 2.

## Figures and Tables

**Table 1:** Geometric mean, 50th and 90th percentiles of selected non-dioxin-like polychlorinated biphenyls (PCBs) concentrations (in ng/g of lipid or parts per billion on a lipid weight) by US Census region, NHANES 2003-2004.

	Ages of 20 years and older		
	Geometric mean (95% CI)	Percentile (95% CI)	
		50th	90th
2,4,4′-Trichlorobiphenyl (PCB 28)			
Northeast	4.1 (3.4–4.9)^a*∗*^	4.1 (3.5–4.7)	7.9 (5.0–13.3)
Midwest	5.1 (3.6–6.6)^a,b^	5.3 (3.7–7.3)	9.0 (7.4–11.3)
South	4.8 (4.1–5.5)^a,b^	4.7 (3.9–5.8)	9.9 (8.4–11.2)
West	5.4 (4.5–6.3)^b^	5.3 (4.5–6.2)	9.5 (7.4–13.6)
2,2′,5,5′-Tetrachlorobiphenyl (PCB 52)			
Northeast	2.3 (1.3–3.3)^a^	2.2 (1.3–3.7)	5.4 (2.3–12.6)
Midwest	2.4 (1.9–2.8)^a^	2.5 (1.9–3.0)	4.9 (3.1–8.9)
South	2.5 (2.0–2.9)^a^	2.6 (2.0–3.2)	6.2 (4.5–7.8)
West	3.5 (3.1–3.9)^b^	3.6 (2.9–4.3)	6.3 (5.2–7.3)
2,4,4′,5-Tetrachlorobiphenyl (PCB 74)			
Northeast	6.6 (4.9–8.3)^b^	5.9 (3.7–9.2)	24.0 (11.8–45.0)
Midwest	5.3 (4.1–6.4)^a^	4.4 (2.9–9.4)	16.0 (9.4–33.9)
South	5.5 (4.6–6.4)^a^	5.8 (3.6–7.9)	17.9 (12.4–22.3)
West	4.5 (3.1–5.9)^a^	4.4 (2.6–7.3)	12.4 (11.5–14.7)
2,2′,4,4′,5-Pentachlorobiphenyl (PCB 99)			
Northeast	6.3 (4.4–8.3)^b^	5.7 (3.2–14.1)	21.1 (8.7–51.2)
Midwest	4.2 (2.7–5.8)^a^	3.8 (2.8–5.4)	12.6 (4.4–79.5)
South	4.6 (3.7–5.5)^a^	4.2 (3.4–5.2)	15.7 (8.8–19.0)
West	3.7 (3.0–4.3)^a^	3.5 (2.9–4.1)	8.7 (6.9–11.5)
2,2′,4,5,5′-Pentachlorobiphenyl (PCB 101)			
Northeast	1.5 (0.84–2.2)^a^	1.5 (0.90–2.4)	5.1 (2.4–10.8)
Midwest	1.4 (1.1–1.8)^a^	1.4 (0.94–2.3)	3.6 (2.5–5.5)
South	1.6 (1.2–1.9)^a^	1.6 (1.4–1.8)	4.5 (3.2–6.8)
West	2.1 (1.8–2.4)^b^	2.1 (1.7–2.6)	4.2 (3.3–5.4)
2,2′,3,3′,4,4′,5,6′-Octachlorobiphenyl and 2,2′,3,4,4′,5,5′,6-octachlorobiphenyl (PCBs 196 and 203)			
Northeast	4.3 (2.6–6.0)^a^	4.7 (3.3–6.5)	16.3 (4.5–80.1)
Midwest	3.2 (2.0–4.3)^b^	3.8 (2.9–4.9)	11.5 (7.3–16.1)
South	3.1 (2.4–3.8)^b^	4.3 (3.0–5.0)	10.9 (9.5–12.1)
West	3.4 (1.8–5.0)^a^	3.7 (1.7–7.7)	12.9 (10.6–16.5)
2,2′,3,3′,4,4′,5,5′,6-Nonachlorobiphenyl (PCB 206)			
Northeast	3.5 (2.4–4.5)^a^	3.3 (2.0–5.6)	13.7 (5.6–41.5)
Midwest	2.5 (1.5–3.5)^b^	2.6 (1.8–3.6)	8.9 (4.9–19.2)
South	2.7 (1.7–3.7)^b^	3.1 (2.0–4.5)	9.7 (7.4–15.7)
West	2.0 (0.83–3.3)^b^	2.0 (0.83–5.3)	7.8 (5.7–11.4)

^*∗*^Regional means with the same letter superscript are not significantly different from each other based on analysis of covariance adjusting for age, smoking status, gender, and race/ethnicity.

**Table 2:** Geometric mean and selected percentiles of serum PCBs 138 and 158 and PCB 153 concentrations (in ng/g of lipid or parts per billion on a lipid weight) and HxCDD and HpCDD concentrations (in pg/g of lipid or parts per trillion on a lipid weight basis) for the US population by geographic area, NHANES 2001–2004.

	Ages of 20 years and older		
	Geometric mean (95% CI)	Percentile (95% CI)	
		50th	90th
2,2′,3,4,4′,5′-Hexachlorobiphenyl and 2,3,3′,4,4′,6-hexachlorobiphenyl (PCBs 138 and 158)			
Northeast	27.4 (25.5–29.2)^a*∗*^	28.1 (25.7–30.9)	88.7 (77.2–104)
Midwest	19.4 (15.4–23.5)^b^	18.8 (15.3–24.5)	64.7 (50.0–88.4)
South	21.0 (18.2–23.7)^b^	22.5 (19.2–25.3)	69.5 (60.5–76.0)
West	17.3 (15.9–18.6)^b^	16.8 (15.2–19.4)	51.8 (47.5–63.0)
2,2′,4,4′,5,5′-Hexachlorobiphenyl (PCB 153)			
Northeast	37.1 (34.2–40.1)^a^	40.5 (36.2–43.7)	118 (95.1–139)
Midwest	26.5 (21.0–32.0)^b^	27.8 (20.4–36.5)	92.4 (66.2–117)
South	28.1 (24.7–31.5)^b^	31.1 (26.7–35.0)	91.3 (78.0–109)
West	24.4 (22.5–26.2)^b^	25.9 (22.3–28.3)	74.3 (68.6–81.3)

1,2,3,6,7,8-Hexachlorodibenzo-p-dioxin (HxCDD)			
Northeast	25.1 (18.5–31.6)^a^	29.8 (24.0–37.3)	73.5 (60.6–89.1)
Midwest	29.6 (19.1–40.1)^a^	34.5 (23.3–48.1)	94.2 (53.1–181)
South	25.7 (21.3–30.1)^a^	29.5 (25.4–35.5)	73.4 (62.3–92.4)
West	22.1 (19.1–25.1)^a^	26.0 (22.0–30.6)	60.7 (52.1–74.2)
1,2,3,4,6,7,8-Heptachlorodibenzo-p-dioxin (HpCDD)			
Northeast	29.4 (23.2–35.7)^a,b^	30.1 (24.8–36.5)	82.2 (56.7–115)
Midwest	35.9 (27.7–44.1)^a^	37.1 (27.3–51.0)	112 (75.6–158)
South	34.1 (29.6–38.7)^a^	35.3 (29.6–41.3)	98.2 (83.7–125)
West	27.1 (24.4–29.7)^b^	28.9 (25.9–31.7)	71.7 (66.1–74.7)

^*∗*^Regional means with the same letter superscript are not significantly different from each other based on analysis of covariance adjusting for age, smoking status, gender, and race/ethnicity.

**Table 3:** Geometric mean and selected percentiles of serum DDE concentrations (in ng/g of lipid or parts per billion on a lipid weight basis) for the US population by geographic area, NHANES 1999–2004.

	Ages of 20 years and older		
DDE	Geometric mean	Percentile (95% CI)	
1999–2004	(95% CI)	50th	90th
Northeast	247 (211–284)^a*∗*^	198 (174–233)	1180 (972–1400)
Midwest	232 (201–263)^a^	195 (175–240)	919 (706–1150)
South	311 (262–360)^b^	260 (227–310)	1480 (1180–1870)
West	476 (390–563)^c^	421 (360–545)	1720 (1440–2090)

^*∗*^Regional means with the same letter superscript are not significantly different from each other based on analysis of covariance adjusting for age, smoking status, gender, and race/ethnicity.

**Table 4:** Geometric mean and percentiles of selected serum PBDE concentrations (in ng/g of lipid or parts per billion on a lipid weight basis) for the US population by geographic area, NHANES 2003-2004.

	Ages of 20 years and older		
	Geometric mean (95% CI)	Percentile (95% CI)	
		50th	90th
2,4,4′-Tribromodiphenyl ether (BDE 28)			
Northeast	0.8 (0.6–0.9)^a*∗*^	0.7 (0.6–0.8)	2.5 (1.4–7.7)
Midwest	1.1 (0.9–1.4)^b^	1.0 (0.9–1.1)	3.5 (2.1–7.2)
South	1.1 (0.9–1.3)^b^	1.1 (0.9–1.4)	4.7 (3.2–6.6)
West	2.1 (0.8–3.5)^c^	2.1 (1.2–3.5)	8.3 (3.9–20)
2,2′,4,4′-Tetrabromodiphenyl ether (BDE 47)			
Northeast	12.5 (7.3–17.8)^a^	11.5 (7.5–17.1)	51.5 (19.7–171)
Midwest	16.9 (12.9–20.8)^a,b^	13.9 (11.0–21.7)	65.4 (47.5–83.3)
South	20.7 (17.0–24.4)^b^	20.4 (16.0–25.4)	84.9 (63.3–121)
West	33.9 (17.6–60.2)^b,c^	29.4 (14.1–79.5)	171 (70.0–589)
2,2′,4,4′,6-Pentabromodiphenyl ether (BDE 100)			
Northeast	2.5 (1.4–3.5)^a^	2.2 (1.2–4.1)	11.1 (4.3–21.2)
Midwest	3.3 (1.7–4.8)^a,b^	2.6 (1.7–5.0)	16.6 (8.3–30.5)
South	4.1 (3.4–4.8)^b,c^	3.9 (3.1–4.9)	17.9 (12.9–24.6)
West	6.0 (2.0–9.9)^b,c^	5.3 (2.9–9.3)	34.5 (8.4–158)
2,2′,4,4′,5,5′-Hexabromodiphenyl ether (BDE 153)			
Northeast	3.9 (2.2–5.6)^a^	3.0 (1.9–5.1)	19.6 (9.4–60.5)
Midwest	4.6 (0.35–8.8)^a,b^	3.8 (1.4–14.1)	24.3 (8.5–140)
South	6.1 (5.6–6.7)^b,c^	4.9 (4.3–5.3)	34.3 (26.2–44.7)
West	7.2 (4.4–9.9)^b,c^	6.1 (3.5–10.6)	62.9 (22.9–88.4)

^*∗*^Regional means with the same letter superscript are not significantly different from each other based on analysis of covariance adjusting for age, smoking status, gender, and race/ethnicity.

**Table 5:** Geometric means and selected percentiles of the sum of 35 PCBs concentrations and total TEQ in ng/g of lipid or parts per billion on a lipid weight basis for the US population by geographic area, NHANES 2003-2004.

	Ages of 20 years and older		
	Geometric mean (95% CI)	Percentile (95% CI)	
		50th	90th
Sum of 35 PCBs			
Northeast	189 (173–204)^a*∗*^	187 (138–250)	534 (346–929)
Midwest	144 (114–175)^b^	142 (85–213)	405 (270–713)
South	152 (123–182)^b^	156 (122–206)	430 (358–484)
West	141 (99–182)^b^	138 (94–208)	406 (287–508)
Total TEQ			
Northeast	—^*∗∗*^	—	35.5 (30.6–41.9)
Midwest	—	—	35.3 (22.7–59.0)
South	—	—	29.7 (25.5–36.4)
West	—	—	28.2 (24.1–32.6)

^*∗*^Regional means with the same letter superscript are not significantly different from each other based on analysis of covariance adjusting for age, smoking status, gender, and race/ethnicity.

^*∗∗*^For TEQ estimates, we present only the 90th percentile because the detection frequency was less than 40% for some congeners included in the calculation.

## Abbreviations

CI: Confidence interval
DDT: Dichlorodiphenyltrichloroethane
DDE: Dichlorodiphenyldichloroethylene
GM: Geometric mean
HxCDD: 1,2,3,6,7,8-Hexachlorodibenzo-p-dioxin
HpCDD: 1,2,3,4,6,7,8-Heptachlorodibenzo-p-dioxin
NCHS: National Center for Health Statistics
NHANES: National Health and Nutrition Examination Survey
PBDEs: Polybrominated diphenyl ethers
BDE 28: 2,4,4′-Tribromodiphenyl ether
BDE 47: 2,2′,4,4′-Tetrabromodiphenyl ether
BDE 100: 2,2′,4,4′,6-Pentabromodiphenyl ether
BDE 153: 2,2′,4,4′,5,5′-Hexabromodiphenyl ether
PCBs: Polychlorinated biphenyls
PCB 28: 2,4,4′-Trichlorobiphenyl
PCB 52: 2,2′,5,5′-Tetrachlorobiphenyl
PCB 101: 2,2′,4,5,5′-Pentachlorobiphenyl
PCB 74: 2,4,4′,5-Tetrachlorobiphenyl
PCB 99: 2,2′,4,4′,5-Pentachlorobiphenyl
PCB 206: 2,2′,3,3′,4,4′,5,5′,6-Nonachlorobiphenyl
PCBs 196/203: 2,2′,3,3′,4,4′,5,6′-Octachlorobiphenyl and 2,2′3,4,4′,5,5′,6-octachlorobiphenyl
PCBs 138 and 158: 2,2′,3,4,4′,5′-Hexachlorobiphenyl and 2,3,3′,4,4′,6-hexachlorobiphenyl
PCB 153: 2,2′,4,4′,5,5′-Hexachlorobiphenyl
PCDDs: Dibenzo-p-dioxins
PCDFs: Polychlorinated dibenzofurans
PFCs: Perfluoroalkyl chemicals
PFHxS: Perfluorohexane sulfonic acid
PFNA: Perfluorononanoic acid
PFOA: Perfluorooctanoic acid
PFOS: Perfluorooctane sulfonic acid
POPs: Persistent organic pollutants
RDC: Research Data Center.
