# Supplementary material for: Regional Variation in Human Exposure to Persistent Organic Pollutants in the United States, NHANES
Source: J Environ Public Health. 2015 Dec 29;2015:571839. doi: 10.1155/2015/571839 (PMC4709617; doi:10.1155/2015/571839)
Supplement: Supplementary file 1 — Supplemental Table 1 provides a summary of the NHANES survey years selected based primarily on availability for each environmental chemical in the current report. Supplemental Table 2 provides the geometric mean concentrations and selected percentiles (in micrograms per liter) of serum perfluorononanoic acid (PFNA), perfluorohexane sulfonic acid (PFHxS), perfluorooctanoic acid (PFOA), and perfluorooctane sulfonic acid (PFOS) for adults ages 20 years and older by U.S. census region for NHANES 2003-2010. These data supplement the regional comparisons based on geometric means that are presented in Figure 2. [file 571839.f1.pdf]

Supplemental Tables

Table S-1. Summary of NHANES Environmental Chemicals and Available Cycles*

**Table/Figure**	**Analytes**	**NHANES Survey Years with at least 60% detection rate**
Table 1	PCB 28, PCB 52, PCB 101, PCB 74, PCB 99, PCB 196/203, PCB 206	2003-2004
Table 2	PCB 138/158, PCB 153, HxCDD, HpCDD	2001-2004
Table 3	pp-DDE	1999-2004
Table 4	PBDEs	2003-2004
Table 5	Sum of 35 PCBs and Total TEQs	2003-2004
Figure 2	PFCs (PFOS, PFNA, PFHxS and PFOA)	2003-2010<br>2 year cycle data are available since 1999; there is a continuous deceasing trend for PFOS, and a continuous increasing trend for PFNA.
*The RDC proposal was submitted in September 2011.		

Table S-2. Geometric mean and selected percentiles of serum perfluorinated compound concentrations (in µg/L) for the U.S. population by geographic area, NHANES 2003-2010			
	Age 20 years and older		
	Geometric mean (95% CI)	Percentile (95% CI)	
		50th	90th
**Perfluorononanoic acid (PFNA)**			
Northeast	1.4 (1.2-1.6)[a,b]	1.4 (1.1-1.8)	2.9 (2.6-3.4)
Midwest	1.2 (1.1-1.2)[a]	1.2 (1.1-1.3)	2.2 (2.0-2.4)
South	1.6 (1.3-1.8)[b]	1.6 (1.4-1.8)	4.2 (3.0-5.9)
West	0.9 (0.8-1.0)[c]	1.0 (0.9-1.1)	2.0 (1.9-2.1)
**Perfluorohexane sulfonic acid (PFHxS)**			
Northeast	1.5 (1.2-1.7)[a]	1.6 (1.2-1.9)	4.2 (3.4-5.2)
Midwest	1.7 (1.5-1.9)[a]	1.7 (1.5-1.9)	5.4 (4.8-5.9)
South	2.1 (1.8-2.4)[b]	2.1 (1.9-2.4)	6.3 (4.8-9.0)
West	1.5 (1.4-1.6)[a]	1.6 (1.4-1.8)	4.2 (3.7-4.5)
**Perfluorooctanoic acid (PFOA)**			
Northeast	3.8 (3.4-4.2)[a,b]	4.0 (3.7-4.4)	7.3 (6.5-8.3)
Midwest	3.6 (3.2-3.9)[a]	3.7 (3.4-4.0)	7.2 (5.8-8.9)
South	4.3 (3.9-4.7)[b]	4.6 (4.2-5.0)	9.6 (7.9-11.6)
West	3.2 (3.0-3.4)[a]	3.5 (3.2-3.7)	6.9 (6.5-7.3)
**Perfluorooctane sulfonic acid (PFOS)**			
Northeast	13.3 (11.4-15.1)[a]	13.7 (11.9-16.0)	30.3 (27.6-33.7)
Midwest	14.2 (13.0-15.3)[a]	15.0 (13.6-16.3)	34.5 (32.3-38.0)
South	17.9 (15.8-20.0)[b]	19.3 (17.1-21.7)	44.7 (38.9-54.6)
West	11.9 (10.7-13.0)[a]	12.6 (11.5-13.9)	29.1 (25.9-32.2)
* Regional means with the same letter superscript are NOT significantly different from each other based on analysis of covariance adjusting for age, smoking status, gender and race/ethnicity.			
